# Factors associated with secondhand smoke exposure in different settings: Results from the German Health Update (GEDA) 2012

**DOI:** 10.1186/s12889-016-3007-z

**Published:** 2016-04-14

**Authors:** Florian Fischer, Alexander Kraemer

**Affiliations:** Department of Public Health Medicine, School of Public Health, Bielefeld University, P.O. Box 100 131, 33501 Bielefeld, Germany

**Keywords:** Secondhand smoke, SHS, German Health Update

## Abstract

**Background:**

The ubiquity of secondhand smoke (SHS) exposure at home or in private establishments, workplaces and public areas poses several challenges for the reduction of SHS exposure. This study aimed to describe the prevalence of SHS exposure in Germany and key factors associated with exposure. Results were also differentiated by place of exposure.

**Methods:**

A secondary data analysis based on the public use file of the German Health Update 2012 was conducted (*n* = 13,933). Only non-smokers were included in the analysis. In a multivariable logistic regression model the factors associated with SHS exposure were calculated. In addition, a further set of multivariable logistic regressions were calculated for factors associated with the place of SHS exposure (workplace, at home, bars/discotheques, restaurants, at the house of a friend).

**Results:**

More than a quarter of non-smoking study participants were exposed to SHS. The main area of exposure was the workplace (40.9 %). The multivariable logistic regression indicated young age as the most important factor associated with SHS exposure. The odds for SHS exposure was higher in men than in women. The likelihood of SHS exposure decreased with higher education. SHS exposure and the associated factors varied between different places of exposure.

**Conclusions:**

Despite several actions to protect non-smokers which were implemented in Germany during the past years, SHS exposure still remains a relevant risk factor at a population level. According to the results of this study, particularly the workplace and other public places such as bars and discotheques have to be taken into account for the development of strategies to reduce SHS exposure.

## Background

Globally, tobacco use is one of the leading preventable causes of morbidity and mortality. Diseases associated with tobacco use pose a significant burden on individuals, societies and healthcare systems. Smoking affects not only active smokers but also those who are exposed to secondhand smoke (SHS) in the vicinity of a smoker [1, 2]. Consistent adverse health effects caused by SHS exposure have been reported [3]. The ubiquity of tobacco smoke at home or in private establishments, workplaces and public areas (indoor and outdoor) poses several challenges to policymakers and society for the reduction of SHS exposure [4].

Bans and policies for tobacco control can be implemented through public health policies or legislation affecting populations at a national, state or community level [3]. A Cochrane review summarizing 77 studies observes consistent positive health effects after the implementation of legislative smoking bans. According to the results of this systematic review, consistent evidence of a positive impact of national smoking bans on improving cardiovascular health outcomes, and reducing mortality for associated smoking-related illnesses exists  [3]. This is also true for strategies focusing on SHS exposure in workplaces [5, 6]. Besides aspects of legislation on smoke-free workplaces and public places, also increased taxes, mass media education, restrictions on tobacco advertising, school-based or community programmes and cessation assistance are considerable options [7–9].

In Germany, the federal law for the protection from the hazards of SHS exposure came into force in September 2007. This led to a ban on smoking in federal facilities as well as constitutional bodies of the federation and in public transport systems (train stations and public transportation services such as airplanes, trains, buses, trams, taxis, etc.). Exceptions are possible for separate and appropriately marked spaces [10]. Furthermore, the federal *Workplace Regulations* were modified so that employers must ban smoking in all or at least specific areas of the workplace [11]. Since 2007, the protection of non-smokers in the gastronomy sector has been regulated by state laws. Until now, the regulation of other indoor public places varies between the federal states with more or less strict regulations, because each of the 16 federal states of Germany has a different set of regulations for the protection of non-smokers [12].

### Aims and objectives

Until now, only few studies aimed to focus on the determinants of SHS exposure, although this information is needed for adequate public health policies to protect non-smokers. Determinants of SHS exposure at the workplace for bar and restaurant workers were in the focus of a recent study performed in Chile [13]. Further studies were mainly performed among Asian populations and/or focused on youths and adolescents [14–20]. Studies on the determinants of SHS exposure in European countries are scarce and were conducted before recent legislations to protect non-smokers were implemented [21, 22]. Therefore, this study aims to describe the prevalence of SHS exposure in the general adult population of non-smokers in Germany, stratified by different subgroups of the population and by different settings where SHS exposure might take place. Furthermore, the most important factors associated with SHS exposure in the German population, also differentiated by settings of exposure, will be assessed. The information provided in this study will allow for the development and implementation of targeted preventive measures for the reduction of SHS exposure.

## Methods

### Study population

The secondary data analysis is based on the public use file of the German Health Update 2012 (GEDA 2012), which is part of the nationwide health monitoring conducted by the Robert Koch Institute. In the German Health Update 2012 a total sample of 19,294 persons 18 years or older participated in Computer Assisted Telephone Interviews (CATI), which is representative for the adult population in Germany. The cross-sectional data were collected between February 2012 and March 2013 [23].

Since the study is based on secondary data analysis, no ethical approval is needed for this analysis. For data collection, the Robert Koch Institute observed the Federal Data Protection Act, which means that all data were collected and analyzed in an anonymous manner [24].

### Variables selected for analysis

The information on the dependent variable was assessed by the question, how many days per week the respondent was exposed to SHS. This question was not asked to smokers. Therefore, the sample size was 13,933 people by including only non-smokers. This sample was used for the descriptive and bivariable analyses to calculate three groups of SHS exposure: No SHS exposure, low SHS exposure (1–3 days per week) and high SHS exposure (4 or more days per week). In the multivariable logistic regression model exposure (low and high SHS exposure combined) was compared to no exposure. In addition, among exposed persons (*n* = 3,820) a further set of multivariable logistic regressions were calculated for factors associated with the place of SHS exposure (“workplace”, “at home”, “bars/discotheques”, “restaurants”, “at the house of a friend”). Therefore, a second set of dependent variables was used, which included information on the place of exposure. The participants were able to declare exposure at more than one place.

The study has an exploratory character. The choice of independent variables was based on previous study results described in the literature as determinants of SHS exposure. Only independent variables being significantly associated with the dependent variable (exposure vs. no exposure) were included in the model. Furthermore, only low levels of correlations between independent variables were allowed, before the variables were selected for the multivariable logistic regression model. The first model aims to assess the factors being associated with SHS exposure. All independent variables selected for the first model were used for the second set of models aiming to describe the factors associated with the place of SHS exposure. The independent variables were taken from the data set of the public use file, except the variable on the place of residence. This variable contained information on population structure types (*Siedlungsstrukturelle Kreistypen*) and was coded with four items. This variable was recoded into two items (“rural” and “urban”). Further independent variables included information on sex (“male” and “female”), age groups (“18–29 years”, “30–44 years”, “45–64 years” and “65 years and more”), socioeconomic status (“low”, “middle” and “high”), educational level (“low”, “middle” and “high” [based on ISCED 1997]), migrant background (“yes” and “no”), living together with a partner (“yes” and “no”), overall physical activity (“<2.5 h per week” and “≥2.5 h per week”) and risky alcohol consumption (“yes” and “no” [based on AUDIT-C]).

### Statistical analysis

Data were analyzed using IBM SPSS Statistics 21. To control for possible selection biases, a weighting factor for the GEDA 2012 sample was provided. This weighting factor was constructed by taking age, sex, educational status and geographical regions into consideration [23]. It was used for all analyses in this study.

The descriptive analysis contained frequency runs to explore information about the sample and SHS exposure. Bivariable analyses in forms of cross-tables were calculated. It was performed by comparing independent variables with the depend variables categorized with three items (no, low and high exposure). We used the chi-square test of independence to analyze the associations between two variables with multiple categories. Statistical significant differences were considered using *p-values* ≤ 0.05 based on two-tailed tests. Correlations between all selected variables (data not shown) and the variance inflation factor (VIF) were calculated in order to test for multicollinearity. Due to a moderate to strong correlation between the socioeconomic status and the educational level (*r* = 0.627), the variable on the socioeconomic status was excluded from the multivariable analyses. After exclusion of the variable on the socioeconomic status, the VIF ranged from 1.005 to 1.134, indicating no multicollinearity.

Odds ratios (OR) and 95 % confidence intervals (CI) were calculated in the multivariable analyses. The first model compared people exposed with people unexposed. Nagelkerke’s R^2^ (0.223) indicated that almost a quarter of the variance can be explained by the independent variables included in the model. The factors associated with SHS exposure described in the first model of the multivariable analysis included only the overall status of exposure as dependent variable. Nevertheless, SHS exposure may occur at different places. Therefore, the second part of the multivariable statistics included only people exposed to SHS to compare factors which are associated with SHS exposure in different settings. In the survey, exposed people were able to mention all settings where they were exposed to SHS. A set of five places for potential SHS exposure was selected (workplace, at home, bars/discotheques, restaurants, at the house of a friend) to illustrate differences in associations of the independent variables and the respective place of SHS exposure.

## Results

### Descriptive analysis

The sample characteristics are described in Table 1. 53.9 % of study participants are female and more than half of the respondents show a medium socioeconomic as well as educational status. Only a very small proportion (*n* = 308; 2.6 %) has a migrant background.

**Table 1:** Sample characteristics and bivariable analysis in the German Health Update (2012)

	Sample characteristics^a^	SHS exposure^a^			
		n (%)			
	n (%)	Never	Low	High	p-value*
			(1–3 days per week)	(4+ days per week)	
*Sex*					
Male	6,424 (46.1)	4,251 (66.2)	1,126 (17.5)	1,047 (16.3)	<0.001
Female	7,509 (53.9)	5,880 (78.3)	869 (11.6)	760 (10.1)	
*Age*					
18–29 years	2,038 (14.6)	852 (41.8)	624 (30.6)	562 (27.6)	<0.001
30–44 years	3,023 (21.7)	1,991 (65.9)	510 (16.9)	522 (17.3)	
45–64 years	4,692 (33.7)	3,498 (74.6)	615 (13.1)	579 (12.3)	
+ 65 years	4,180 (30.0)	3,790 (90.7)	246 (5.9)	144 (3.4)	
*Socioeconomic status*					
Low	2,792 (20.1)	1,974 (70.7)	322 (11.5)	496 (17.8)	<0.001
Middle	8,114 (58.3)	5,779 (71.2)	1,225 (15.1)	1,110 (13.7)	
High	3,003 (21.6)	2,362 (78.7)	444 (14.8)	197 (6.6)	
*Educational status*					
Low	2,808 (20.2)	2,027 (72.2)	314 (11.2)	467 (16.6)	<0.001
Middle	7,430 (53.4)	5,177 (69.7)	1,166 (15.7)	1,087 (14.6)	
High	3,671 (26.4)	2.912 (79.3)	510 (13.9)	249 (6.8)	
*Migrant background*					
Yes	308 (2.6)	165 (53.6)	60 (19.5)	83 (26.9)	<0.001
No	11,521 (97.4)	8,335 (72.3)	1,717 (14.9)	1,469 (12.8)	
*Place of residence*					
Urban	9,451 (67.8)	6,816 (72.1)	1,375 (14.5)	1,260 (13.3)	0.064
Rural	4,482 (32.2)	3,315 (74.0)	620 (13.8)	547 (12.2)	
*Living together with partner*					
Yes	9,028 (65.1)	6,955 (77.0)	1,038 (11.5)	1,035 (11.5)	<0.001
No	4,850 (34.9)	3,141 (64.8)	946 (19.5)	763 (15.7)	
*Physical activity (hours per week)*					
≥2.5 h	5,237 (38.2)	3,498 (66.8)	842 (16.1)	897 (17.1)	<0.001
<2.5 h	8,458 (61.8)	6,440 (76.1)	1,131 (13.4)	887 (10.5)	
*Risky alcohol consumption*					
Yes	3,213 (23.3)	2,124 (66.1)	616 (19.2)	473 (14.7)	<0.001
No	10,606 (76.7)	7,923 (74.7)	1,369 (12.1)	1,314 (12.4)	

^a^Sample sizes may not add up to 13,933 due to missing values; weighted results

*p-value based on two-tailed Pearson’s *χ* ^2^ test

Overall, 72.7 % of respondents claimed not to be exposed to SHS, 14.3 % were exposed 1 to 3 days per week and further 13.0 % were exposed 4 or more days per week. Among the 3,820 people exposed to SHS, the main area of exposure was the workplace (*n* = 1,562; 40.9 %), followed by exposure at the house of a friend (*n* = 1,280; 33.5 %). About a quarter of respondents declared exposure in bars/discotheques (*n* = 1,002; 26.2 %) or at home (*n* = 957; 25.1 %). Only a small proportion reported SHS exposure in restaurants (*n* = 255; 6.7 %).

### Bivariable analysis

The bivariable analysis revealed higher prevalences of SHS exposure in males compared to females. Furthermore, high SHS exposure was more frequent with younger age. People with high socioeconomic status as well as high educational level showed the lowest prevalences of high SHS exposure (4+ days per week). High SHS exposure was more frequent in migrants than in non-migrants (26.9 % vs. 12.9 %). There were no significant differences for SHS exposure between rural and urban areas. Study participants living with a partner showed lower prevalences of SHS exposure. People with a higher level of physical activity and people with risky alcohol consumption had higher proportions of SHS exposure (Table 1).

### Multivariable analysis: Factors associated with SHS exposure

The results of this model presented in Table 2 compare people exposed to SHS (low and high exposure combined) to the reference group of unexposed people. According to these results, age is the most important factor associated with SHS exposure. People aged 18–29 years showed an OR of 11.38 (95 % CI: 9.67–13.41) compared to people aged 65 years and more. With increasing age the likelihood of SHS exposure decreased. The OR for SHS exposure was higher in men (OR = 1.91; 95 % CI: 1.75–2.10) than in women and higher in people with migrant background (OR = 1.38; 95 % CI: 1.06–1.78). The likelihood of SHS exposure decreases with higher education. This is indicated by the result that the likelihood of reporting SHS exposure among people with low educational status was 2.13 (95 % CI: 1.83–2.47) times that of people with higher education.

**Table 2:** Multivariable analysis – Factors associated with SHS exposure in the German Health Update (2012)

	*B*	*Std. Error*	*Wald*	*OR*	*95 % CI*	p-value*
Intercept	−3.17	0.10	1,019.21			
*Sex*						
Male	0.65	0.05	194.91	1.91	1.75–2.10	<0.001
Female (ref.)				1.00		
*Age*						
18–29 years	2.43	0.08	850.66	11.38	9.67–13.41	<0.001
30–44 years	1.79	0.08	528.99	5.97	5.12–6.95	<0.001
45–64 years	1.26	0.07	301.69	3.53	3.06–4.07	<0.001
+ 65 years (ref.)				1.00		
*Educational status*						
Low	0.75	0.08	94.57	2.13	1.83–2.47	<0.001
Middle	0.60	0.06	119.88	1.83	1.64–2.03	<0.001
High (ref.)				1.00		
*Migrant background*						
Yes	0.32	0.13	5.87	1.38	1.06–1.78	0.015
No (ref.)				1.00		
*Place of residence*						
Urban	0.17	0.04	12.51	1.19	1.08–1.31	<0.001
Rural (ref.)				1.00		
*Living together with partner*						
Yes	−0.29	0.06	27.79	0.75	0.67–0.83	<0.001
No (ref.)				1.00		
*Physical activity (hours per week)*						
≥2.5 h	0.16	0.05	12.09	1.18	1.07–1.29	0.001
<2.5 h (ref.)				1.00		
*Risky alcohol consumption*						
Yes	0.30	0.05	34.47	1.35	1.22–1.50	<0.001
No (ref.)				1.00		

Reference category: not exposed; weighted results

*p-value based on two-tailed Pearson’s *χ* ^2^ test

Although not statistically significant in the bivariable analysis, living in urban areas leads to a slightly but significantly increased odds of being exposed to SHS (OR = 1.19; 95 % CI: 1.08–1.31) compared to rural areas. Living together with a partner has a protective effect (OR = 0.75; 95 % CI: 0.67–0.83) compared to living alone. People with risky alcohol consumption had a 35 % higher odds to be exposed to SHS (OR = 1.35; 95 % CI: 1.22–1.50) than people without risky alcohol consumption. In contrast, the positive health behavior of frequent physical activity was associated with a higher likelihood of SHS exposure (OR = 1.18; 95 % CI: 1.07–1.29).

### Multivariable analysis: Factors associated with places of SHS exposure

The results of these five separated multivariable analyses, according to place of SHS exposure, are described in Table 3. The results indicate major differences. First of all, the variances of SHS exposure at the different places which can be explained by the selected independent variables differ largely. For the workplace as well as bars/discotheques, almost 18 % of the variance can be explained, as shown by Nagelkerke’s R^2^. This is much lower for SHS exposure at home (9.8 %), at the house of a friend (5.8 %) or in restaurants (3.6 %). Therefore, the variables selected are insufficient to explain the factors associated with SHS exposure at places like restaurants, at home or at the house of a friend. Nevertheless, the variables are quite useful for the workplace, where most people (40.9 %) were exposed to SHS.

**Table 3:** Multivariable analysis – Factors associated with places of SHS exposure in the German Health Update (2012)

	*Workplace (n = 1,562; 40.9 %)*	*At home (n = 957; 25.1 %)*	*Bars/discotheques (n = 1,002; 26.2 %)*	*Restaurants (n = 255; 6.7 %)*	*At the house of a friend (n = 1,280; 33.5 %)*
*Sex*					
Male	2.42 (2.06–2.83)**	0.41 (0.35–0.49)**	1.39 (1.16–1.65)**	1.10 (0.82–1.47)	0.73 (0.63–0.85)**
Female (ref.)					
*Age*					
18–29 years	8.32 (5.06–13.67)**	0.50 (0.37–0.68)**	3.35 (2.34–4.79)**	0.52 (0.32–0.84)*	1.13 (0.85–1.51)
30–44 years	15.75 (9.68–25.61)**	0.41 (0.30–0.55)**	1.62 (1.13–2.33)*	0.68 (0.36–0.92)*	1.16 (0.87–1.54)
45–64 years	12.80 (7.90–20.73)**	0.44 (0.33–0.58)**	1.42 (0.99–2.02)	0.47 (0.29–0.76)*	0.69 (0.52–0.91)*
+ 65 years (ref.)					
*Educational status*					
Low	1.18 (0.91–1.52)	2.10 (1.59–2.76)**	0.39 (0.30–0.52)**	0.32 (0.19–0.55)**	0.91 (0.71–1.16)
Middle	1.49 (1.23–1.80)**	1.21 (0.96–1.51)	0.67 (0.54–0.82)**	0.84 (0.60–1.17)	0.96 (0.79–1.16)
High (ref.)					
*Migrant background*					
Yes	1.76 (1.22–2.53)*	1.58 (1.08–2.31)*	0.70 (0.45–1.09)	1.94 (1.09–3.46)*	1.23 (0.87–1.75)
No (ref.)					
*Place of residence*					
Urban	0.90 (0.76–1.05)	0.93 (0.78–1.12)	1.27 (1.06–1.52)*	1.43 (1.03–1.98)*	1.18 (1.00–1.39)*
Rural (ref.)					
*Living together with partner*					
Yes	1.22 (1.01–1.48)*	1.62 (1.31–2.00)**	0.55 (0.45–0.67)**	0.62 (0.44–0.86)*	0.63 (0.52–0.75)**
No (ref.)					
*Physical activity (hours per week)*					
≥2.5 h	1.22 (1.05–1.42)*	1.12 (0.95–1.33)	0.81 (0.68–0.96)*	1.06 (0.80–1.42)	0.99 (0.85–1.16)
<2.5 h (ref.)					
*Risky alcohol consumption*					
Yes	0.72 (0.61–0.85)**	0.80 (0.66–0.97)*	2.73 (2.30–3.25)**	1.05 (0.78–1.43)	1.32 (1.12–1.55)*
No (ref.)					
*Nagelkerke’s R* ^*2*^	0.177	0.098	0.177	0.036	0.058

Reference category: not exposed; weighted results

Values represent odds ratios and 95% confidence intervals

* *p* < 0.05; ** *p* < 0.001

In addition to the differences in explained variance also the direction of associations varied between the places of SHS exposure. For example, in workplaces and bars/discotheques males were more likely to be exposed to SHS, whereas at home and at the house of a friend women were more likely to be exposed to SHS. In contrast to the results of the regression model focusing on the overall SHS exposure, the results for different places of exposure for the educational level were not that clear. Furthermore, several variables that were significant in the overall multivariable model lose their significance when the regression is stratified by the place of residence.

## Discussion

With more than one quarter of the study population being exposed to SHS, our study reveals the large impact of this risk factor at population level. SHS exposure occurs in several settings: at home, in the workplace, and in other indoor places such as restaurants and bars [25, 26], as well as public (outdoor) places [27]. Therefore, programmes and legislation have to be setting specific. This is strengthened by the result of this study that the factors associated with SHS exposure differ considerably between places of exposure.

Several studies from different world regions have already highlighted the aspect of higher SHS exposure in younger age groups [28–30], which was also the main factor associated with overall exposure in this analysis. Regarding the level of exposure stratified by sex, the overall likelihood of being exposed to SHS was higher in men than in women. Nevertheless, this is dependent on the place of exposure. This is also consistent with previous findings, where SHS exposure was more prevalent at home in women [31] and more prevalent at the workplace in men [32]. These differences emphasize the urgent need to assess the SHS exposure in a more detailed and specific way, instead of relying on the sole information whether exposure was existent or not.

The higher likelihoods of SHS exposure in people with low socioeconomic status and low level of education revealed in this study are comparable with previous literature [28, 33, 34]. Furthermore, the higher likelihood of migrants being exposed to SHS was already described in studies from the USA [32]. A further study conducted in Germany showed that children with a migration background had an increased risk of SHS exposure in their homes [35].

The comparatively low prevalence of people exposed in restaurants (6.7 %) might be due to the legislative protection of non-smokers in the gastronomy sector which was implemented in Germany in 2007, but also due to the fact that the a priori likelihood of being exposed is higher in workplaces and at home, because people do not visit restaurants so frequently. Measurements after the implementation of the smoking ban in hospitality venues in Germany showed a decrease in the median mass concentration of respirable suspended particles (PM_2.5_) of 90.8 % in discotheques, 88.7 % in restaurants, 87.5 % in coffee bars and 66.3 % in bars. Despite this significant decline in exposure to SHS in hospitality venues, the German smoke-free legislation was judged to be a mixed success, because all federal states allow exemptions from smoking bans. Therefore, a greater reduction would have been possible by implementing a comprehensive smoking ban without any exemptions [36].

Workplace regulations enforce employers to ban smoking in all or at least specific areas of the workplace in Germany. Nevertheless, the workplace is still a setting which allows for further improvements in the reduction of SHS exposure. This is confirmed by our study results which indicate that 40.9 % of people being exposed to SHS are exposed at the workplace. Positive effects of smoking bans in workplaces have been described for non-smokers, because these strategies have led to substantial reductions in cardiovascular diseases for people being exposed to SHS. Non-smokers who are no longer exposed to SHS realize 60 % of the benefit and smokers who quit smoking realize 40 % of the benefit of reductions in tobacco consumption. This is due to the larger number of people being exposed to SHS compared to active smokers [37].

Policies to restrict smoking in public places are necessary for several reasons. First of all, the majority of the public experiences annoyance and discomfort from SHS exposure and judges this exposure to be hazardous to health. Nevertheless, most non-smokers do not take personal action to avoid SHS exposure. To protect non-smokers, actions by official sites are necessary [38]. Furthermore, restricting smoking in public settings may increase the likelihood that smokers smoke less or quit smoking entirely [39–41]. Some studies indicated that a widespread prohibition of smoking in workplaces and public places makes the home the principal location for SHS exposure in non-smokers [42–44]. Therefore, not only legislations are needed, but also further public health efforts to increase awareness of the risks associated with SHS exposure [45]. If health risks are adequately communicated and oriented towards the demands of (several) target groups, the social acceptability of smoking will further decline [46]. This study highlights that particularly men, young people, and people with low socioeconomic or educational level need to be targeted. These groups need further protection from SHS exposure but also smoking prevention and cessation programmes need to focus on this group. In addition, a focus on the exposure setting is important to develop and establish suitable strategies for the reduction of SHS exposure.

### Limitations

The study has some limitations which have to be acknowledged. Secondary data were taken from a cross-sectional study. Therefore, only variables already included in the data set could be used for the analysis, although further variables might also have been relevant to find factors associated with SHS exposure. Furthermore, due to the study design independent and dependent variables were measured at a single point in time. For that reason no causal relationships could be drawn. In addition, the effects of legislation could only be supposed and not proven by this analysis. The variables were self-reported, which may lead to misclassifications due to recall and reporting bias. Even though the results particularly for the measurement of exposure might be biased, they are more likely to result in underreporting than overreporting. Because the assessment of exposure was based on self-reported data, only crude categories (exposed vs. not exposed) were chosen for the multivariable analysis, to avoid any false precision in terms of exposure categories.

The second part of the multivariable analysis contained just those variables already selected for the regression aiming to describe the factors with overall SHS exposure. Therefore, these factors should be interpreted with caution. For example, all non-smokers were included in the analysis, irrespective of their employment situation. Therefore, significant associations might partly reflect factors associated with being employed rather than only those associated with being exposed to SHS at the workplace. It was intended to highlight differences between the factors associated with SHS exposure in these settings, but not to describe the best model explaining most of the variance of SHS exposure in each of the five selected places of exposure.

## Conclusion

Despite several actions to protect non-smokers which were implemented in Germany during the past years, SHS exposure remains a relevant risk factor at population level. SHS exposure varies significantly between different places of exposure. According to the results of this study, particularly the workplace and other public places such as bars and discotheques, but also the home of non-smokers living together with smokers, have to be taken into account for the development of strategies to reduce SHS exposure. Therefore, not only legislations should be implemented, but also further public health strategies must be considered to increase the awareness of adverse health effects caused by SHS exposure. To develop suitable strategies to reduce the SHS exposure, several determinants have to be considered, which differ between the places of exposure.
